# High-resolution climate modelling of fasciolosis risk in Australia: A One Health early-warning framework

**DOI:** 10.1016/j.onehlt.2026.101451

**Published:** 2026-05-26

**Authors:** Rana M. Athar Ali, Mark A. Stevenson, Leah Tyrell, Nichola E.D. Calvani, Travis Beddoe, Grant Rawlin, Terry Spithill, Neil D. Young, Abdul Jabbar

**Affiliations:** a Melbourne Veterinary School, The University of Melbourne, Victoria, Australia; b The Mackinnon Project, The University of Melbourne, Victoria, Australia; c Sydney School of Veterinary Science, Faculty of Science, The University of Sydney, New South Wales, Australia; d Department of Ecological, Plant and Animal Sciences and Centre for AgriBioscience, La Trobe University, Victoria, Australia; e Agriculture Department of Energy, Environment and Climate Action, Victoria, Australia

**Keywords:** Climate change, Liver fluke, Predicting risk, Australia

## Abstract

Fasciolosis, caused by the liver fluke *Fasciola hepatica*, is a climate-sensitive parasitic disease that threatens livestock health, productivity and farm profitability, with broader implications for food security, rural livelihoods, and sustainable food systems. The disease is endemic in Victoria, Australia, where environmental and climatic conditions favour parasite transmission at the livestock–environment interface. This study used high-resolution (5 km^2^) climate data from 40,504 grid points across Victoria to validate a Growing Degree Days (GDD) model for estimating fasciolosis risk over the past 50 years (1975–2024) and to project future risk for 2050 and 2090 under medium (RCP 4.5) and high (RCP 8.5) representative concentration pathways. Linear regression analysis identified a strong relationship between observed fasciolosis prevalence and modelled risk values (R^2^ = 0.94, *p* < 0.003). Historical data analyses showed substantial interannual variability, with consistently higher risk in eastern Victoria, followed by the western and northern regions. Under future climate scenarios, fasciolosis risk increased spatially 2-fold in Barwon and 3-fold in the Great South Coast, particularly under the RCP 8.5 scenario in 2090. Risk remained sustained in Gippsland, especially in southern areas, covering 35% of the region, while a modest increase (5%) was projected for Greater Melbourne relative to a baseline risk of 45%. These findings highlight important One Health implications, as climate-driven changes in fasciolosis risk may undermine livestock productivity, and challenge the resilience of livestock-derived food systems. The study provides an evidence base to support climate-responsive surveillance, risk-based control strategies, and integrated animal health and environmental policy development.

## Highlights

• Fasciolosis is endemic in Victoria, Australia and is strongly climate driven.
• GDD model accurately predicted fasciolosis risk across Victoria.
• Eastern Victoria showed highest historical fasciolosis risk.
• Climate change may double–triple risk in key dairy regions.
• Risk maps support targeted, climate-smart liver fluke control.

## Graphical abstract

**Figure:**
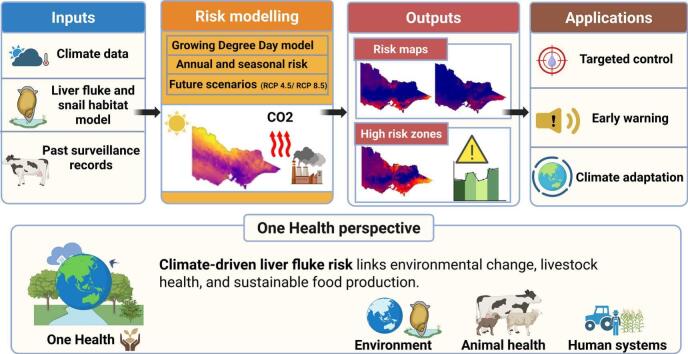

## 1 Introduction

Fasciolosis, caused by the liver flukes *Fasciola hepatica* and *Fasciola gigantica*, is a globally important parasitic disease that threatens livestock health and productivity, contributing to substantial economic losses [1], [2]. Beyond its direct impacts on animal health and farm profitability, fasciolosis has broader One Health relevance through its effects on food security, sustainable livestock systems, climate-sensitive agroecosystems and its zoonotic implications. Similar to other environmentally mediated parasitic diseases, the prevalence and spatial distribution of fasciolosis are strongly influenced by climatic and environmental conditions, particularly mean temperatures above 10 °C, high rainfall, adequate moisture, and the presence of water bodies [3]. These factors govern the development and survival of the free-living stages of *F. hepatica* and its intermediate freshwater snail host belonging to the family Lymnaeidae [3]. Irrigation practices further modify local hydrology and microclimates, making it challenging to determine the spatial distribution of endemic lymnaeid snail populations and liver fluke infections in livestock, particularly in Victoria, Australia [4], [5].

Globally, a range of modelling approaches have been developed to predict liver fluke distribution and risk, including ecological niche, mechanistic, growing degree day (GDD), and GIS-based models [6], [7], [8], [9], [10], [11]. GDD models estimate the timing of key biological processes by calculating accumulated heat units from maximum and minimum temperature data and form the foundation of many fasciolosis prediction frameworks [6], [7], [12], [13], [14]. Among these, the Ollerenshaw index was one of the earliest and most widely applied models, used in the United Kingdom and elsewhere to simulate long-term risk of fasciolosis and guide livestock management decisions [15], [16], [17]. However, both original and modified versions of the Ollerenshaw index have shown declining predictive ability in recent years, with discrepancies between predicted and observed prevalence and evidence of risk underestimation in the UK [18]. Similarly, validation efforts in New South Wales, Australia, demonstrated poor correlation between Ollerenshaw index predictions and local prevalence data, underscoring its limited suitability for Australian climatic conditions [19].

More recently, the GDD-based model developed by Malone et al. [14] has been successfully applied across diverse ecological and climatic settings to evaluate *F. hepatica* distribution. This approach predicted substantial increases in contracting liver fluke infection in the Canterbury and Otago regions of New Zealand [20]. It also demonstrated clear seasonal transmission patterns and climate sensitivity for *F. hepatica* infection in Iran [21]. Despite the well-recognised role of climate in shaping the risk of fasciolosis, no model has yet been validated or implemented in Australia that integrates historical and projected climate data to assess long-term risk dynamics.

In Victoria, the southeastern state of Australia, early studies of *F. hepatica* prevalence relied primarily on visual inspection of livers at abattoirs [22], [23]. More recent investigations using sensitive diagnostic tools, including faecal egg counts and a commercial coproantigen ELISA, have identified a markedly higher prevalence, reaching 80% in the Macalister Irrigation District compared with the Goulburn and Upper Murray regions [5], [20]. While these cross-sectional studies provided limited insight into seasonal dynamics of *F. hepatica* infections, environmental surveillance has shown higher counts of *F. hepatica* and *Austropeplea tomentosa* in water samples during autumn and spring, consistent with earlier observations of seasonal peaks in infection risk [3], [25], [26].

In response to increasing concern over climate change, the Australian Climate Science Centre of the Commonwealth Scientific and Industrial Research Organisation (CSIRO) have developed high-resolution downscaled climate datasets derived from global climate models (GCMs), including temperature, rainfall, and potential evapotranspiration, for various Australian states (e.g., Victoria) [27]. These datasets were generated for two representative concentration pathways, RCP 4.5 (medium greenhouse gas emissions) and RCP 8.5 (high emissions), providing a robust basis for future climate impact assessments [27].

This study represents the first Australian effort to quantify the geographic distribution of fasciolosis risk over the past 50 years and to project future risk for near (2050) and far (2090) time horizons using climate-driven modelling. A One Health framework was applied by integrating climatic and environmental variables, including temperature thresholds, rainfall, potential evapotranspiration, and snail habitat suitability indices, within a GDD modelling approach. By linking climate change with parasite development, snail ecology, and livestock exposure, this study provides critical insights into future expected shifts in fasciolosis risk across Victoria. The findings provide an early-warning tool for livestock producers, their advisors and policymakers, supporting climate-responsive surveillance, targeted control strategies, and adaptive management to protect livestock productivity, food systems, and environmental health under changing climatic conditions.

## 2 Materials and methods

### 2.1 Study area

Victoria, located in southeast Australia, is one of the country's major livestock-producing states, supporting dairy, beef, wool, and mixed agricultural industries. The state has the largest dairy herd and contributes more than 62% of Australia's milk production and ranks third nationally for beef production [28]. Victoria is bordered by New South Wales to the north and South Australia to the west, with its southern coastline along the Bass Strait separating it from Tasmania. The state encompasses diverse climatic zones and landscapes, ranging from high rainfall regions to extensive lowland water bodies, irrigation districts, and water channels. These conditions support year-round grazing systems but also create environments conducive to environmentally mediated parasitic infections, including fasciolosis, at the livestock–environment interface.

### 2.2 Climate data

#### 2.2.1 Historical climate data

Daily Australian climate datasets for maximum temperature (T_max_), minimum temperature (T_min_), rainfall (R), and potential evapotranspiration (PET) for the period 1 January 1975 to 31 December 2024 were obtained from the Australian Bureau of Meteorology via the SILO database^1^ in R v.4.4.1 (R Core Team, 2025) using the contributed *httr*, *jsonlite*, and *ncdf4* packages [29], [30], [31]. Each dataset comprised gridded rasters at a spatial resolution of 0.05° × 0.05° (approximately 5 km × 5 km), representing 18,250 daily raster quartets (365 days × 50 years). All data were georeferenced using the WGS84 coordinate reference system (EPSG:4362).

**Fig. 1:**
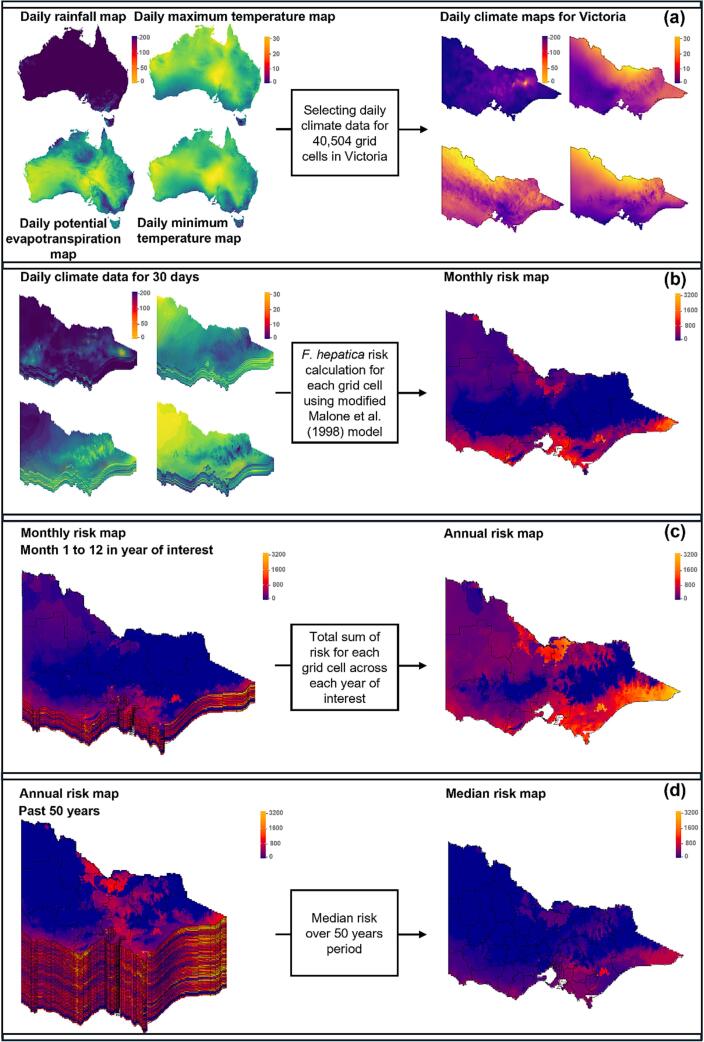
Overview of the data processing and modelling steps used to derive (a) daily climatic and environmental variables, (b) calculate monthly and (c) annual fasciolosis risk and (d) produce median risk maps for Victoria during the 50-year study period (1975–2024).

Each of the climate variable raster maps were clipped using a vector map of the state boundary of Victoria. Spatial data on declared irrigation regions were obtained from the Victorian Department of Energy, Environment and Climate Action (GIS Help Desk). For each day, mean temperature (T_mean_) was calculated as the average of daily T_min_ and daily T_max_. Daily T_min_, T_max_, T_mean_, PET, and R (Fig. 1a) estimates for each raster cell were aggregated to monthly means for T_min_, T_max_ and T_mean_ and monthly totals for PET and R. The number of wet days per month (rainfall >1 mm) was also derived. Monthly temperature, PET and rainfall raster layers were used to calculate monthly fasciolosis risk estimates (Fig. 1b) using the methodology of Malone et al. [14], described in detail below.

Monthly risk maps were then averaged across their respective year to return annual risk maps (Fig. 1c). Each of the 50 annual risk maps were then combined to derive an overall median risk surface for Victoria (Fig. 1d).

#### 2.2.2 Climate change projection data

High-resolution daily climate simulation datasets (in NetCDF format) for 2050 and 2090 were obtained from the *Climate Change in Australia* database (Victorian Climate Projections 2019; accessed 01 July 2025). These datasets corresponded to two greenhouse gas emission scenarios, Representative Concentration Pathways RCP 4.5 (medium emissions) and RCP 8.5 (high emissions) and comprised 8640 daily raster quartets derived from six host global climate models (GCMs) used in the Victorian Climate Projections framework [27].

To minimise individual model bias and capture cross-model climatic variability relevant to impact assessment, a multi-model mean approach was applied across the six GCMs [27]. Similar to the approach taken for the historical climate data, monthly means for T_max_, T_min_, PET, and R were calculated for each grid cell by averaging outputs across all models. These downscaled datasets were developed to support climate impact and adaptation studies and enable comparability across emission scenarios and time horizons.

### 2.3 Fasciola risk calculation

The risk of *Fasciola hepatica* infection was estimated using a modified Growing Degree Days (GDD)-based model originally developed by Malone et al. [14]. This mechanistic modelling approach integrates biologically relevant climatic drivers, including temperature, rainfall, potential evapotranspiration, and irrigation, that influence parasite development, snail habitat suitability, and transmission at the livestock–environment interface. Compared with correlative modelling approaches, the GDD framework provides biologically interpretable estimates of seasonal and spatial risk, particularly suited for use with climate change projection data.

The model comprises two components: (1) estimation of average monthly GDD under conditions of available soil moisture in the upper 2.5 cm of soil; and (2) estimation of GDD during periods of monthly soil water surplus [14]. Monthly risk, f, was calculated as:

(1) $$f=\left\{\begin{array}{c} \mathrm{GDD}\times D+\left(\mathrm{GDD}\times \mathrm{WD}\right)\;\frac{\left(\mathrm{RI}-\mathrm{PET}\right)}{25}\;\mathrm{if}\;\mathrm{RI}-\mathrm{PET}>0\\ \mathrm{GDD}\times D,\mathrm{if}\;0<\mathrm{RI}-0.8\times \mathrm{PET}\leq 0\\ 0,\mathrm{otherwise} \end{array}\right)$$

Where:

GDD: average monthly mean temperature (°C) - 10.

D: days in a month.

R: total monthly rainfall (mm).

PET: potential evapotranspiration (mm per month).

WD: number of wet days per month (i.e., days on which rainfall >1 mm).

I: irrigation factor (1 for non-irrigated areas, 2 for irrigated areas).

Fluke risk categories were defined as negligible risk (<600 units), low risk (601 to 1500 units), medium risk (1501 to 3000 units), and high risk (>3000 units), following Yilma and Malone [32]. Monthly rather than annual climate data were used to capture seasonal dynamics relevant to parasite and snail ecology in Victoria, with the exact number of days per month used to avoid underestimation of risk values.

Given the documented influence of irrigation on *F. hepatica* transmission in Victoria [4], an irrigation factor was incorporated by multiplying rainfall by I, reflecting the role of both natural and anthropogenic water sources in creating suitable snail habitats. Grid cells outside declared irrigation regions were assigned a value of one, while grid cells within irrigated regions were assigned a value of two. All analyses were conducted at a spatial resolution of 5 km × 5 km across Victoria.

To support interpretation and decision-making under climate uncertainty, interannual variability in historical risk (1975 to 2024) and cross-model variability in future projections were summarised at regional scales, providing context for robust, risk-based surveillance and adaptive disease management.

### 2.4 Data distribution and regional summaries

Data distributions were assessed for normality using descriptive statistics and visual inspection of histograms. Both historical and future risk datasets showed deviation from normality and contained high-risk outliers. Consequently, a log_10_ transformation was applied prior to statistical analysis. Regional summaries were generated by aggregating grid-level risk estimates within local government areas (Supplementary Table 1) which were grouped into 10 high-resolution regions. Separate risk estimates were compiled for irrigated regions. Median values and interquartile ranges (25th and 75th percentiles) were calculated (Supplementary Tables 2 and 3).

### 2.5 Model validation

Model validation was performed using herd-level true prevalence data for *F. hepatica* in dairy cattle from six irrigated regions (n = 6) in Victoria [5]. Corresponding median modelled risk values were extracted for the same regions and year. The observed prevalence of fasciolosis was treated as the response variable, and modelled risk values were treated as explanatory variables. Linear regression was used to quantify the association between predicted fasciolosis risk and observed prevalence.

### 2.6 Reproducibility and data availability

All data processing, modelling, and statistical analyses were conducted using R v.4.4.1 (R Core Team, 2025) with reproducible workflows consistently applied across historical and future climate datasets. Historical climate data were obtained from the SILO database, and future climate projections were sourced from the *Climate Change in Australia* platform (Victorian Climate Projections 2019). Spatial boundary files and irrigation region datasets were obtained from the Department of Energy, Environment and Climate Action, Victoria. The GDD model was implemented following published formulations [14]. Derived risk maps and regional summaries were generated at a 5 km × 5 km spatial resolution. All scripts used for data extraction, processing, and risk modelling, together with aggregated outputs supporting the findings of this study, are available from the corresponding author upon reasonable request.

## 3 Results

### 3.1 Spatial and temporal mapping of fasciolosis risk

**Fig. 2:**
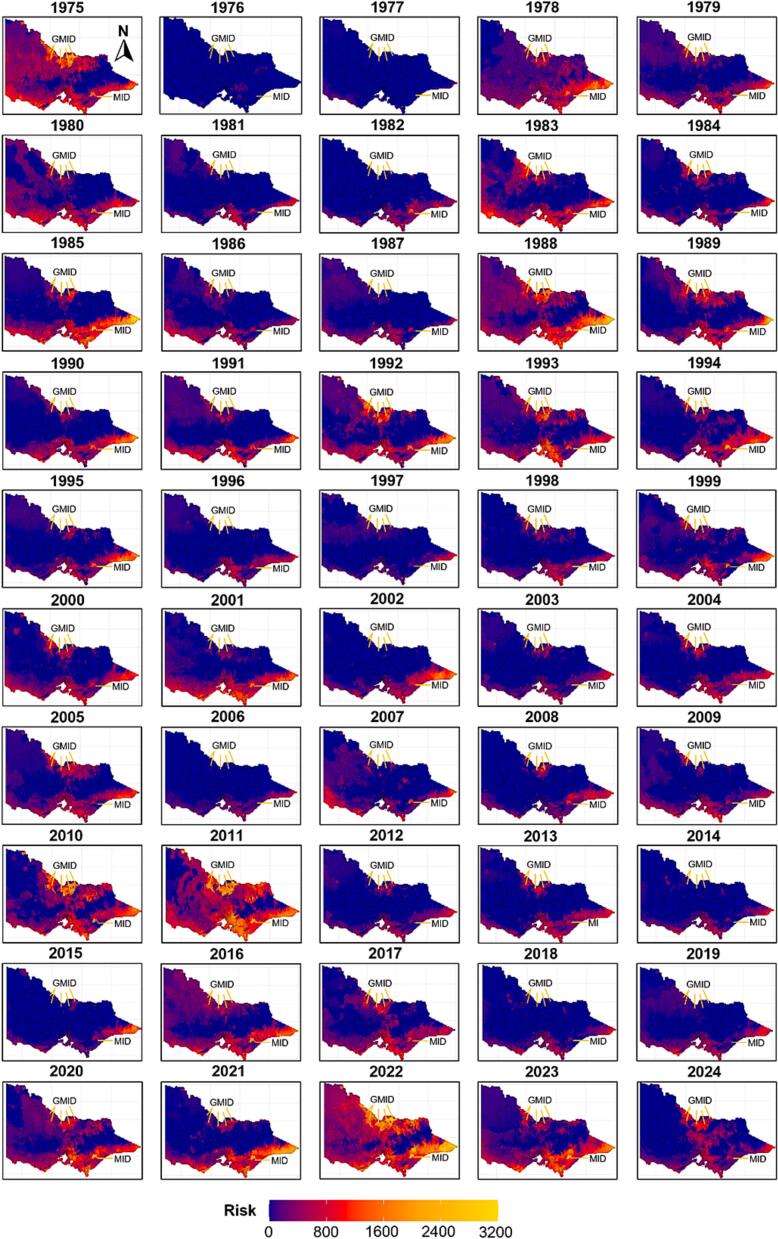
Annual modelled distribution of fascioliasis risk across Victoria, Australia, illustrating spatial and temporal variations over the 50-year study period (1975–2024). A colour gradient represents various risk levels, varying from blue (no risk) to red, orange and yellow (high risk). MID corresponds to Macalister Irrigation District while GMID represents Goulburn Murray Irrigation District. (For interpretation of the references to colour in this figure legend, the reader is referred to the web version of this article.)

The monthly risk of fasciolosis in Victoria was modelled and mapped for the 50-year period from January 1975 to December 2024 (Fig. 2). In total, 600 monthly risk maps were generated, each comprised of 40,504 raster cells uniformly distributed across the state. Each grid cell represented the cumulative fasciolosis risk at a specific longitude–latitude location when the mean temperature exceeded 10 °C and other climatic and environmental criteria of the Malone et al. [14] model (Eq. (1)) were satisfied.

**Fig. 3:**
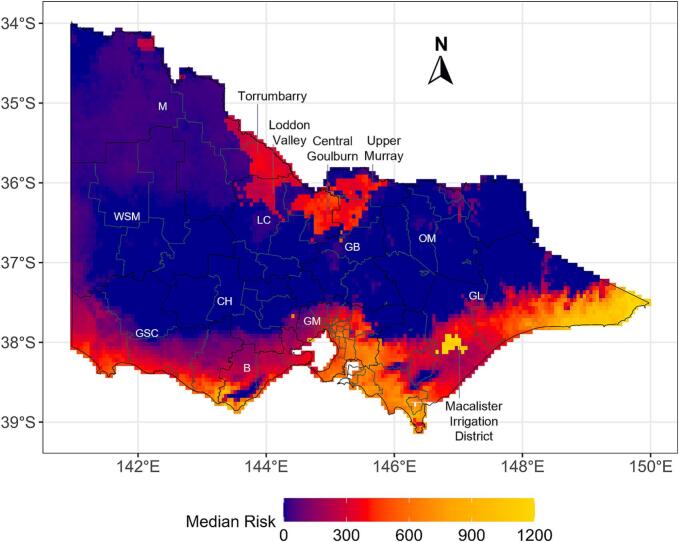
The median risk of *Fasciola hepatica* infection over the 50-year study period (1975–2024). The colour gradient represents various risk levels, ranging from blue (no risk) to red, orange and yellow (high risk). Abbreviations: GL, Gippsland; GM, Greater Melbourne; B, Barwon; GSC, Great South Coast; LC, Loddon Campaspe; GB, Goulburn; OM, Ovens Murray; WSM; Wimmera Southern Mallee; M, Mallee. (For interpretation of the references to colour in this figure legend, the reader is referred to the web version of this article.)

A multi-decade median risk map for the entire study period is shown in Fig. 3. This approach enabled the identification of persistent hotspots and areas of consistently low risk across Victoria. Over the past five decades, fasciolosis risk exhibited marked interannual variability across most regions, reflecting fluctuations in key climatic drivers. An exception was observed in the Macalister Irrigation District, where only minor temporal changes were evident. No consistent long-term trends, either increasing or decreasing, were detected in any region.

Spatially, the highest median risk was concentrated in southeastern Victoria, particularly in Gippsland, including the Macalister Irrigation District, where climatic conditions favoured parasite persistence through higher rainfall, suitable temperatures, and the availability of wet habitats for the intermediate snail host. However, a relatively low risk was identified in northern Victoria, including the Goulburn–Murray irrigation district, across areas such as Torrumbarry, the Loddon Valley, and the Murray Valley. Although southwestern Victoria was generally warmer and drier, parts of the Great South Coast and the Barwon region showed a consistently high fluke risk.

### 3.2 Distribution of transformed risk data

Exploratory analyses of historical and projected risk datasets identified strong right-skewness, with most values clustered at lower risk levels and a long tail of high-risk values. Frequency histograms confirmed substantial deviation from normality. Log10 transformation reduced skewness, resulting in more symmetrical distributions and attenuating the influence of extreme values (Supplementary Fig. S1–S3).

Following the transformation, historical risk data exhibited a single dominant density peak (Fig. S1), indicating relatively homogeneous spatial risk patterns. In contrast, future projections displayed increasing distributional complexity. By 2050, risk values formed two distinct peaks under both emission scenarios (Fig. S2), while by 2090, three peaks were evident (Fig. S3). This progressive shift from unimodal to multimodal distributions indicates increasing spatial and temporal heterogeneity in fasciolosis risk under future climatic conditions across Victoria.

### 3.3 Model validation

**Fig. 4:**
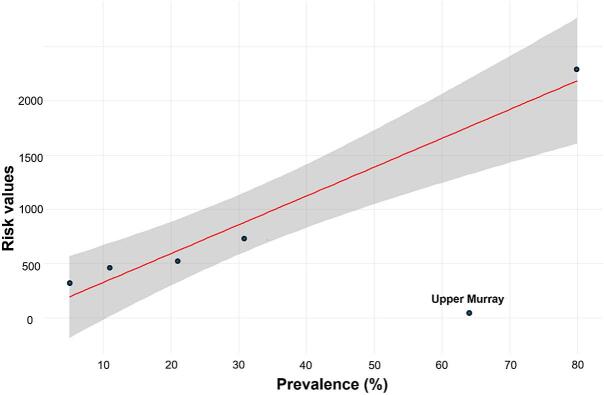
Linear regression model showing the association between observed prevalence and calculated fasciolosis risk across multiple regions in Victoria. The prevalence on the horizontal axis represents the frequency of fasciolosis in each of the six irrigated regions of Victoria. The shaded area represents the 95% confidence interval for the predicted *Fasciola* risk estimates.

Modelled fasciolosis risk showed a strong linear relationship with observed herd-level prevalence of *F. hepatica* in dairy cattle (R^2^ = 0.94, *p* = 0.003). Regions classified as high risk by the model showed higher reported prevalence, while regions with lower predicted risk showed lower prevalence (Fig. 4). One outlier was identified in the Upper Murray region, where high prevalence was recorded despite relatively low predicted risk.

### 3.4 Seasonal patterns of fasciolosis risk

Monthly risk maps from the 50-year dataset were grouped by season (autumn, winter, spring, and summer) to generate median seasonal risk maps. This approach allowed visualisation of consistent seasonal patterns while minimising the influence of interannual variability and extreme values.

**Fig. 5:**
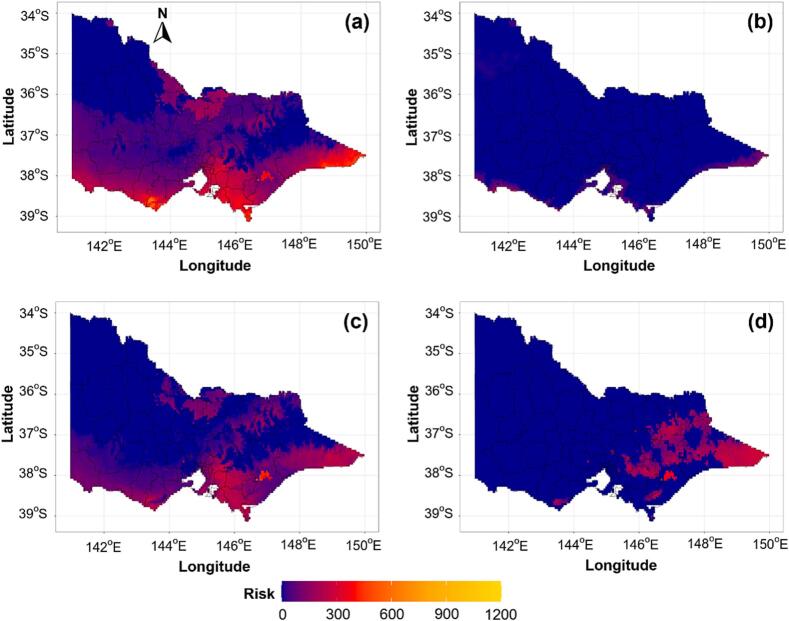
Median seasonal distribution of fasciolosis risk across Victoria, Australia, over the 50-year study period (1975–2024) where a, b, c and d represent autumn (March–May), winter (June–August), spring (September–November) and summer (December–February) seasons, respectively. The colour gradient represents various risk levels, ranging from blue (no risk) to red, orange and yellow (high risk). (For interpretation of the references to colour in this figure legend, the reader is referred to the web version of this article.)

Fasciolosis risk was highest and most widespread during autumn (March–May), particularly in southeastern, southwestern, and northern regions of Victoria (Fig. 5a). Risk declined during winter (June–August) which showed the lowest overall risk, persisting only at low levels in eastern, southeastern and southwestern areas (Fig. 5b). In spring (September–November), risk re-emerged across eastern, southwestern, and parts of northern Victoria (Fig. 5c). Summer (December–February) exhibited a lower overall risk than autumn and spring, largely restricted to eastern regions, consistent with warm and dry seasonal conditions (Fig. 5d). Risk persisted in Macalister Irrigation District among all irrigated regions across three seasons such as autumn, spring and summer (Fig. 5a, c, d). A supplementary map of irrigation areas is shown in Fig. S4.

### 3.5 Projected risk of fasciolosis under different climate change scenarios

**Fig. 6:**
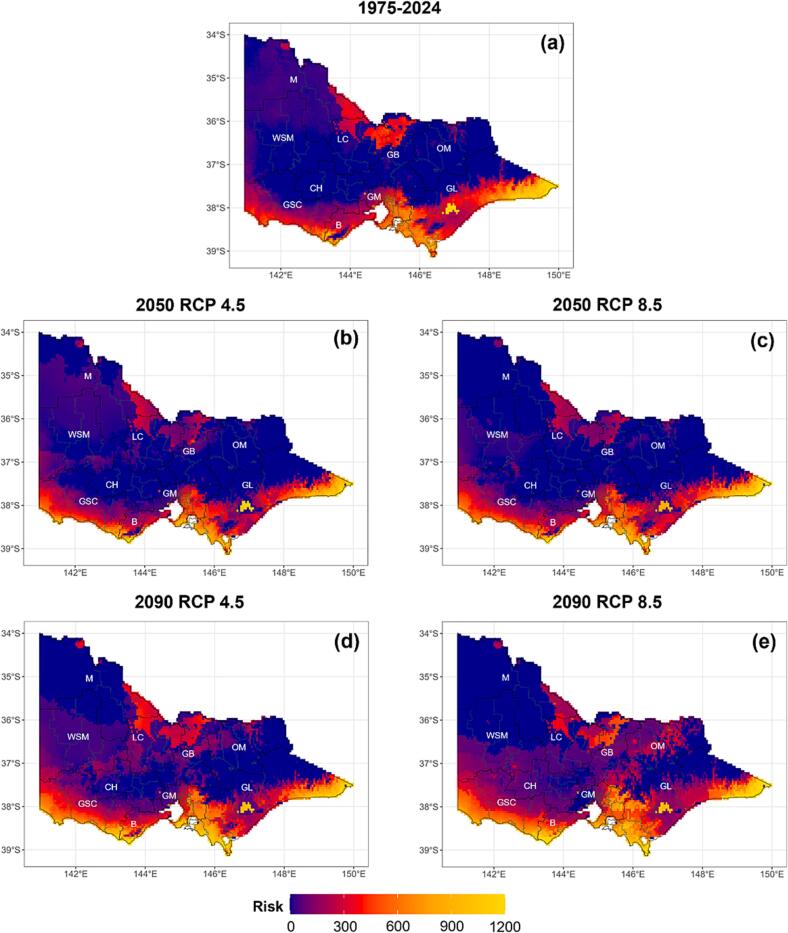
Comparison of fasciolosis risk between the baseline period (1975–2024) and future projections across high-resolution regions in Victoria, Australia, under representative concentration pathways, RCPs for 2050 and 2090. Low emission scenarios are denoted (b) and (d) while high emission scenarios are denoted (c) and (e). The colour gradient represents various risk levels, varying from blue (no risk) to red, orange and yellow (high risk). Abbreviations: GL, Gippsland; GM, Greater Melbourne; B, Barwon; GSC, Great South Coast; LC, Loddon Campaspe; GB, Goulburn; OM, Ovens Murray; WSM; Wimmera Southern Mallee; M, Mallee. (For interpretation of the references to colour in this figure legend, the reader is referred to the web version of this article.)

Median risk values from the historical period (1975 to 2024) were used as the baseline for comparison with projected risks for 2050 and 2090 under medium (RCP 4.5) and high (RCP 8.5) emission scenarios (Fig. 6). Changes in risk area indicate that *F. hepatica* and its intermediate host respond consistently to climate-driven shifts (Table 1).

**Table 1:** Percentage distribution and area of coverage for the risk of fasciolosis in Victoria under baseline (1975–2024) and future projected climate change scenarios in 2050 and 2090.

Regions	1975–2024		2050 RCP 4.5		2050 RCP 8.5		2090 RCP 4.5		2090 RCP 8.5	
	% Distribution	Area (km^2^)	% Distribution	Area (km^2^)	% Distribution	Area (km^2^)	% Distribution	Area (km^2^)	% Distribution	Area (km^2^)
Gippsland	35	15,100	25	10,625	29	12,050	31	12,900	35	15,100
Greater Melbourne	45	4050	37	3350	34	3050	44	3975	50	4500
Barwon	19	1200	19	1225	19	1225	37	2375	48	3075
Great South Coast	12	2875	22	5075	25	5825	34	8050	41	9575
Loddon, Campaspe	7	1275	0	0	0	0	3	725	3	725
Goulburn	7	1225	1	25	0	0	2	300	14	2225
Mallee	0	0	0	0	0	0	2	550	0.1	2.5
Central Highlands	1	25	1	25	1	25	1	25	1	25
Ovens, Murray	0	0	0	0	0	0	0	0	1	25
Wimmera, Southern Mallee	0	0	0	0	0	0	0	0	0	0
Victoria		25,750		20,325		22,175		28,900		35,253

Note: RCP: Representative concentration pathways.

In Gippsland, the baseline risk area of 15,100 km^2^ (35%) decreased to 10,625 km^2^ (25%) under RCP 4.5 and 12,050 km^2^ (29%) under RCP 8.5 by 2050. By 2090, risk areas increased to 12,900 km^2^ (31%) under RCP 4.5 and 14,600 km^2^ (35%) under RCP 8.5. In Greater Melbourne, risk declined from 4050 km^2^ (45%) at baseline to 3350 km^2^ (37%) under RCP 4.5 and 3050 km^2^ (34%) under RCP 8.5 by 2050, before decreasing slightly to 3975 km^2^ (44%) under RCP 4.5 and increasing to 4500 km^2^ (50%) under RCP 8.5 by 2090.

Barwon and the Great South Coast showed pronounced increases in future risk, particularly for the high emissions scenario in 2090. In Barwon, risk increased from 1200 km^2^ (19%) at baseline to 1225 km^2^ (19%) under both scenarios in 2050, before approximately doubling under RCP 4.5 (2375 km^2^; 37%) and almost tripling under RCP 8.5 (3075 km^2^; 48%) by 2090. In the Great South Coast, risk expanded sharply from 2875 km^2^ (12%) at baseline to 5075 km^2^ (22%) under RCP 4.5 and 5825 km^2^ (25%) under RCP 8.5 by 2050, and further increased about 3 fold to 8050 km^2^ (34%) and 9575 km^2^ (41%) under RCP 4.5 and RCP 8.5, respectively, by 2090.

No risk was projected for Loddon Campaspe and Goulburn in 2050 under either scenario; however, by 2090, Loddon Campaspe showed risk across 725 km^2^ (3%) under both emission scenarios and Goulburn increased about 2 fold under RCP 8.5 to 2,225 km^2^ (14%). Risk also emerged in the Mallee by 2090, covering 550 km^2^ (2%) under RCP 4.5 and 2.5 km^2^ (0.1%) under RCP 8.5. At the state level, total risk area declined from 25,750 km^2^ at baseline to 20,325 km^2^ (RCP 4.5) and 22,175 km^2^ (RCP 8.5) by 2050, before increasing substantially to 28,900 km^2^ under RCP 4.5 and 35,253 km^2^ under RCP 8.5 by 2090.

**Fig. 7:**
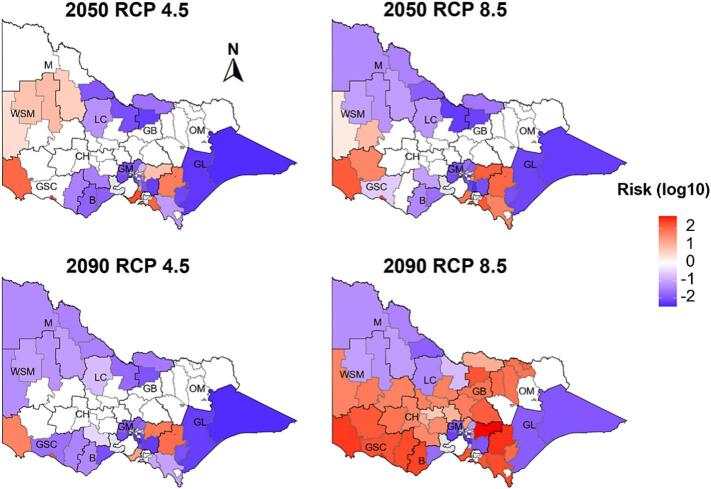
Divergent plots showing projected spatial changes in fasciolosis risk in each Local Government Area located within 10 high-resolution regions of Victoria, Australia, under RCP 4.5 and RCP 8.5 emission scenarios for 2050 and 2090. Red colour gradient indicates increased, blue colour intensity indicates decreased and white indicates no change in risk relative to the historical baseline (1975–2024). Abbreviations: GL, Gippsland; GM, Greater Melbourne; B, Barwon; GSC, Great South Coast; LC, Loddon Campaspe; GB, Goulburn; OM, Ovens Murray; WSM; Wimmera Southern Mallee; M, Mallee. (For interpretation of the references to colour in this figure legend, the reader is referred to the web version of this article.)

Projected changes in fasciolosis risk were further examined at the Local Government Area level within high-resolution regions. Median future risk values for each shire or city were subtracted from historical baselines, log10-transformed, and visualised using divergent plots to illustrate the magnitude and direction of change (Fig. 7). Projections identified marked spatial heterogeneity, with both increases and decreases observed across Victoria. The magnitude of change intensified from 2050 to 2090, particularly under RCP 8.5. Under RCP 4.5 in 2050, moderate risk was observed in eastern Greater Melbourne, parts of South Gippsland, and western Victoria, including areas of the Great South Coast, Mallee, and Wimmera/Southern Mallee, while declines occurred in East Gippsland and northern regions. In 2050 under RCP 8.5, risk increased substantially in eastern Greater Melbourne and South Gippsland, with moderate increases in western regions, including the Great South Coast and Wimmera Southern Mallee, and declines in northern and north-western areas, such as Goulburn, the Mallee and Wimmera Southern Mallee. By 2090, RCP 4.5 projections resembled 2050 patterns with further declines in north-western Victoria, whereas under RCP 8.5, substantial risk increases were predicted across eastern Greater Melbourne, South Gippsland, and western Victoria, including Wimmera Southern Mallee, Barwon and Great South Coast, with an apparent increase in northern regions such as Goulburn.

## 4 Discussion

This study provides the first climate-driven, spatially explicit assessment of historical and future fasciolosis risk across Victoria, Australia, demonstrating how long-term climatic variability and projected climate change are likely to reshape parasite transmission at the animal–environment interface. Using high-resolution climate datasets incorporating minimum and maximum temperature, rainfall, potential evapotranspiration, and irrigation, we applied a biologically informed GDD framework based on the Malone model, originally developed in Louisiana [7] and subsequently applied in East Africa [14], to quantify past and projected risk at a 5 km^2^ spatial resolution. This approach identified pronounced spatial heterogeneity, strong seasonal structure, and region-specific risk trajectories under different emission scenarios. Agreement between modelled risk and herd-level fluke prevalence estimates reported by Kelley et al. [5] provides support for the predictive ability of the model and its use as a decision-support tool for livestock health management. Projected expansion and redistribution of fasciolosis risk in regions such as Barwon, the Great South Coast, Wimmera Southern Mallee, Gippsland, and Greater Melbourne underscore emerging challenges for livestock productivity, sustainable food systems, and climate adaptation planning. Consistent with applications of GDD-based models to other climate-sensitive parasitic diseases of animal and human importance, including dirofilariasis and schistosomiasis [33], [34], [35], these findings reinforce a One Health perspective in which climate-sensitive parasitic diseases are dynamic outcomes of interacting environmental, agricultural, and management systems, requiring integrated surveillance, anticipatory control strategies, and climate-informed policy responses.

In this study, the Malone et al. [14] model was adapted for Australian conditions to enable fasciolosis risk prediction across Victoria. Unlike the Ollerenshaw index, which relies primarily on rainfall and temperature to generate a generalised risk index [12], the Malone model integrates both snail habitat suitability and parasite development indices, providing a more mechanistic and biologically grounded representation of fasciolosis dynamics. Temperature remains the primary determinant of *F. hepatica* and intermediate host occurrence; however, additional climatic factors, including moisture availability and evapotranspiration, play critical roles in parasite and snail survival. Once mean temperatures exceed 10 °C, rainfall, potential evapotranspiration, and irrigation become influential in determining transmission risk. The integration of high-resolution climate datasets (5 km^2^) enabled regional-scale mapping capable of supporting evidence-based disease control, biosecurity planning, and climate adaptation strategies for Victoria.

Over the past five decades, substantial spatial and temporal variability in fasciolosis risk has been observed across Victoria. Although Haydock et al. [20] reported some variations in liver fluke risk from 1972 to 2012 in New Zealand, their findings revealed that the flukey areas remained comparatively stable across various regions in the past. However, we found greater fluctuations across Victoria in from 1975 to 2024, with a flukey region sometimes appearing non-flukey and vice versa. This likely reflects Victoria's greater climatic heterogeneity and sensitivity to large-scale climate drivers, including the El Niño–Southern Oscillation, Southern Annular Mode, Interdecadal Pacific Oscillation, and Indian Ocean Dipole [36], [37]. Similar climate-driven fluctuations in fasciolosis have been reported in northwest Colombia, where La Niña events altered disease transmission dynamics [38].

Retrospective risk mapping identified consistent spatial patterns across Victoria, identifying regions where targeted interventions are likely to be most effective. Risk remained persistently high in parts of Gippsland in southeast Victoria (Fig. 3), where higher autumn and winter rainfall in response to increased thunderstorm activity [39] may promote favourable conditions for snail survival. Long-standing irrigation infrastructure further enhances snail habitat availability [40], and recent environmental surveys have confirmed the presence of the lymnaeid snail host in irrigation channels in this region [26]. Within Gippsland, the Macalister Irrigation District was identified as a persistent fasciolosis risk hotspot over the 50-year period, which is consistent with reports of high (80%) true prevalence of *F. hepatica* in dairy cattle from this area [5], [24]. Comparable irrigation-driven effects on liver fluke transmission have also been reported in Punjab, Pakistan [41].

In southwest Victoria, fasciolosis risk was largely restricted to coastal areas, likely reflecting prolonged dry conditions and declining rainfall that limit snail habitat development further inland [42]. Rainfall in this region is primarily driven by frontal systems and cyclones, which have declined in frequency in recent decades [39], [42]. The absence of declared irrigation systems in this region further constrains snail persistence. Nevertheless, historical surveys reporting liver fluke prevalence of 20–30% along the southwest coast of Victoria [23] support the presence of residual coastal risk (Fig. 3), with episodic above-average rainfall likely enabling transient transmission.

In northern Victoria, including the Goulburn, Loddon Campaspe, and Mallee regions, fasciolosis risk was generally lower and less persistent. Despite lower rainfall compared with southeast Victoria [39], fasciolosis has been reported in dairy cattle in the Goulburn and Loddon Campaspe regions [5], indicating that climatic factors alone do not fully explain disease occurrence. Much of northern Victoria lies within the Goulburn–Murray Irrigation System, where anthropogenic water sources may extend snail habitats beyond naturally suitable environments. However, recent upgrades to irrigation infrastructure, with approximately 80% of dairy producers modernising on-farm systems, may reduce snail populations and associated disease risk [43].

Model validation using coproantigen ELISA-based prevalence data from six irrigated regions provided a robust assessment of active infection [5]. In contrast to abattoir-based inspection data used in some previous studies [20], coproantigen ELISA offers greater sensitivity for detecting current infections [44]. The strong linear relationship observed between modelled risk and observed prevalence (R^2^ = 0.94) indicates that the adapted Malone model reliably reflects regional herd-level infection patterns. The identified outlier in the Upper Murray region may reflect extensive irrigation from the Murray River and several small streams sourced from the Indi and other freshwater springs, which supports snail habitats despite relatively low rainfall [5], [45], a phenomenon also reported in dry zones of New Zealand [20].

Seasonal analyses showed that the highest risk of fasciolosis occurs in autumn, followed by spring, which is consistent with earlier Australian studies [3], [19], [25], [46]. Reduced evapotranspiration in late autumn increases moisture availability, promoting metacercarial survival and infection risk [46]. Longitudinal studies in New South Wales similarly reported seasonal peaks in fasciolosis incidence linked to pasture contamination [3]. Future projections indicate that fasciolosis risk is likely to persist or increase in specific regions under climate change, including sustained risk in Gippsland and Greater Melbourne and marked increases in Barwon (two-fold) and the Great South Coast (three-fold) under high-emission scenarios by 2090. Comparable climate-driven expansions have been reported in New Zealand [20] and Europe [17], underscoring the global relevance of these findings.

In addition to climate change, non-climatic factors such as flooding, farm management practices, limited diagnostic uptake by producers, livestock access to water bodies, and flukicide use patterns all influence fasciolosis prevalence [3], [47]. Treatment frequency, timing, and drug efficacy contribute to the emergence of triclabendazole resistance, which may be further exacerbated by livestock movement, introductions of untreated livestock, and wildlife liver fluke reservoirs [48].

These findings support a shift toward region- and season-specific, risk-based strategies for fasciolosis control. Retrospective and projected risk maps (Fig. 2, Fig. 5, Fig. 6, Fig. 7) provide a practical framework for veterinarians and animal health advisors to tailor surveillance and intervention timing, reducing unnecessary dewormer treatments and mitigating anthelmintic resistance. Policymakers, industry bodies, and animal health professionals should prioritise improved surveillance using sensitive diagnostics to support strategic control under future climate scenarios.

Several limitations should be acknowledged when interpreting these findings. First, the risk of fasciolosis was estimated using a climate-driven GDD framework that captures key biological processes but does not explicitly account for all management-related factors influencing transmission, such as grazing practices, livestock movement, anthelmintic use, or farm-level biosecurity measures. Second, while irrigation was incorporated as a modifying factor, fine-scale hydrological features, soil type variability, and microhabitat conditions that influence snail abundance were not explicitly modelled. Third, future risk projections are inherently subject to uncertainty arising from climate model structure and emission pathway assumptions; although a multi-model mean approach was used to reduce individual model bias, residual uncertainty remains, particularly at local scales. Model validation relied on herd-level prevalence data from a limited number of irrigated regions, which may not fully capture variability across other production systems and landscapes. Finally, while fasciolosis has recognised zoonotic potential, this study focused on livestock and environmental risk and did not explicitly assess human infection risk or food-chain exposure pathways. Addressing these limitations through integrated surveillance, finer-scale environmental data, and cross-sectoral datasets will further strengthen One Health risk assessments under climate change.

## 5 Conclusion

This study provides the first climate-driven, spatially explicit assessment of past and future fasciolosis risk in Victoria, Australia, identifying pronounced spatial and temporal variability associated with climatic conditions. For the period 1975 to 2024, *F. hepatica* risk has remained highest in southeast Victoria, followed by the southwest and northern regions and is projected to increase in specific regions under different high greenhouse gas emission scenarios. These findings support the adoption of risk-based preventive strategies that allow herd managers and their advisors to optimise anthelmintic use by limiting unnecessary treatments in low-risk regions and seasons. Such approaches enhance biosecurity, improve disease management, and support the long-term sustainability of livestock production systems. By integrating climatic and environmental drivers with livestock health outcomes, this work contributes to a proactive One Health framework for managing climate-sensitive parasitic diseases.

## CRediT authorship contribution statement

Rana M. Athar Ali: Writing – original draft, Writing – review & editing, Visualisation, Validation, Software, Resources, Methodology, Investigation, Formal analysis, Data curation, Conceptualisation. Mark A. Stevenson: Writing – review & editing, Software, Methodology, Formal analysis, Supervision, Conceptualisation. Neil D. Young: Writing – review & editing, Visualisation, Validation, Funding acquisition, Conceptualisation, Supervision. Leah Tyrell: Writing – review & editing; Data curation, Supervision. Nichola E.D. Calvani: Writing – review & editing, Visualisation, Validation, Supervision. Travis Beddoe: Writing – review & editing, data curation. Grant Rawlin: Writing – review & editing, data curation, Conceptualisation. Terry Spithill: Writing – review & editing, data curation, Conceptualisation. Abdul Jabbar: Writing – review & editing, Validation, Supervision, Resources, Project administration, Methodology, Investigation, Funding acquisition, Formal analysis, Conceptualisation.

## Ethics approval

Not applicable.

## Funding

This work was supported by the Livestock Biosecurity Funds Grant Program (CCF25.18 to A.J) from Agriculture Victoria and the Victorian Cattle Compensation Fund and a Future Fellowship (FT230100559 to N.D.Y.) from the 10.13039/501100000923Australian Research Council (ARC).

## Declaration of competing interest

The authors declare that they have no known competing financial interests or personal relationships that could have appeared to influence the work reported in this paper.

## Data Availability

Data will be made available on request.

## Footnotes

1 URL: www.longpaddock.qld.gov.au/silo/. Accessed 1 March 2025.
