# Supplementary material for: High-resolution climate modelling of fasciolosis risk in Australia: A One Health early-warning framework
Source: One Health. 2026 May 26;22:101451. doi: 10.1016/j.onehlt.2026.101451 (PMC13227242; doi:10.1016/j.onehlt.2026.101451)
Supplement: Supplementary file 1 — Supplementary material 1 [file mmc1.docx]

**Supplementary figures**

**
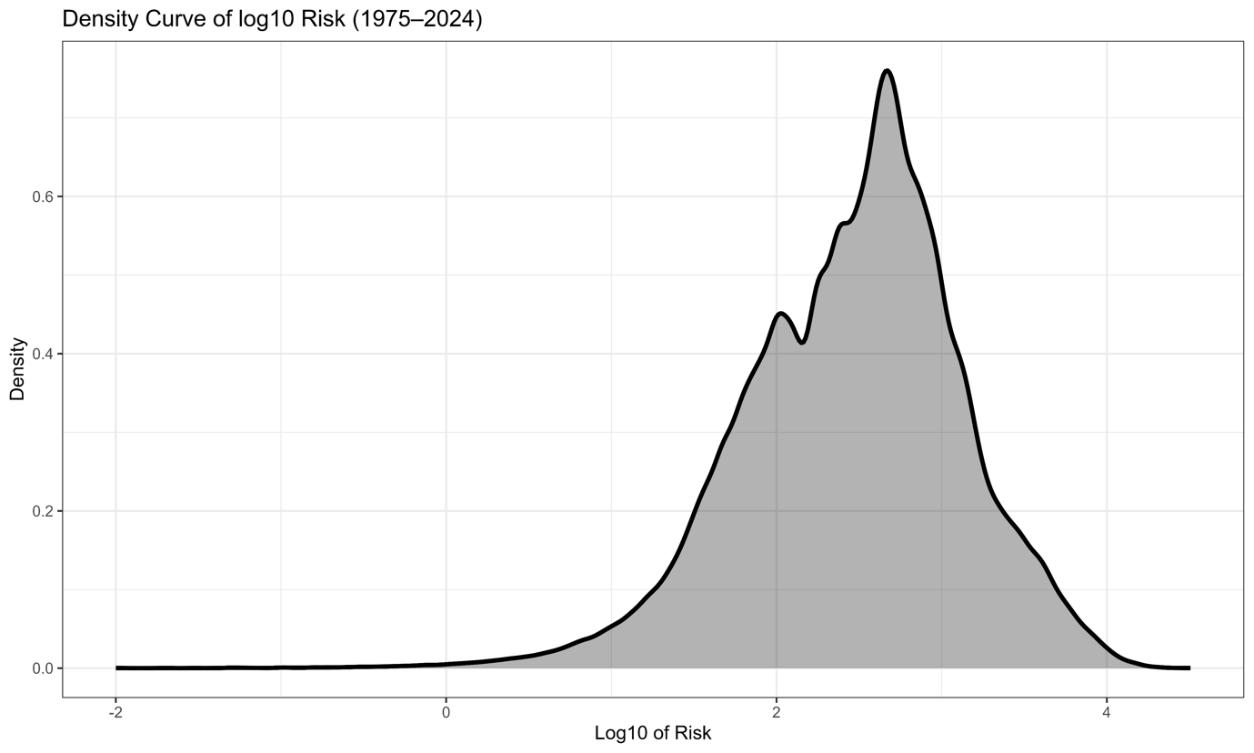
**

**Fig. S1.** Density plot showing the distribution of log₁₀-transformed fasciolosis risk values across Victoria over the 50-year study period (1975–2024). The x-axis represents transformed risk values while the y-axis represents the density of grid points.


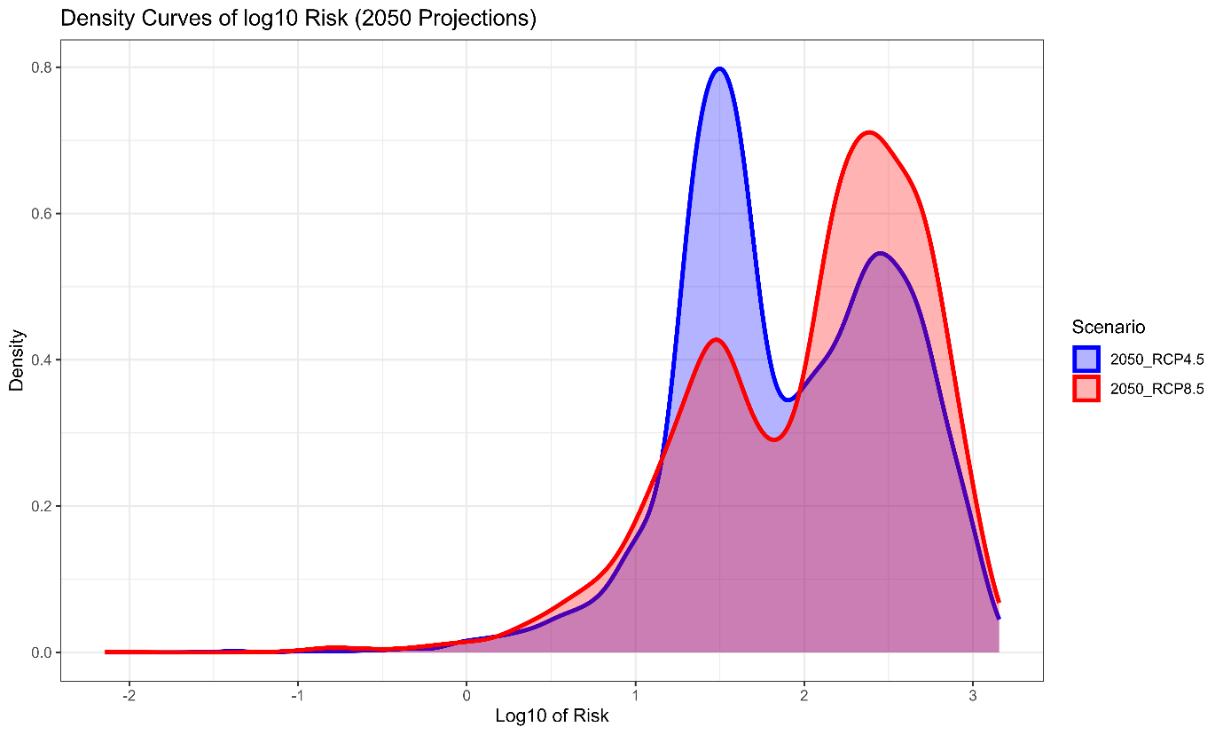


**Fig. S2.** Density plot showing the distribution of log₁₀-transformed fasciolosis risk values across Victoria under RCP 4.5 and RCP 8.5 emission scenarios for the year 2050. The x-axis represents transformed risk values while the y-axis represents the density of grid points.


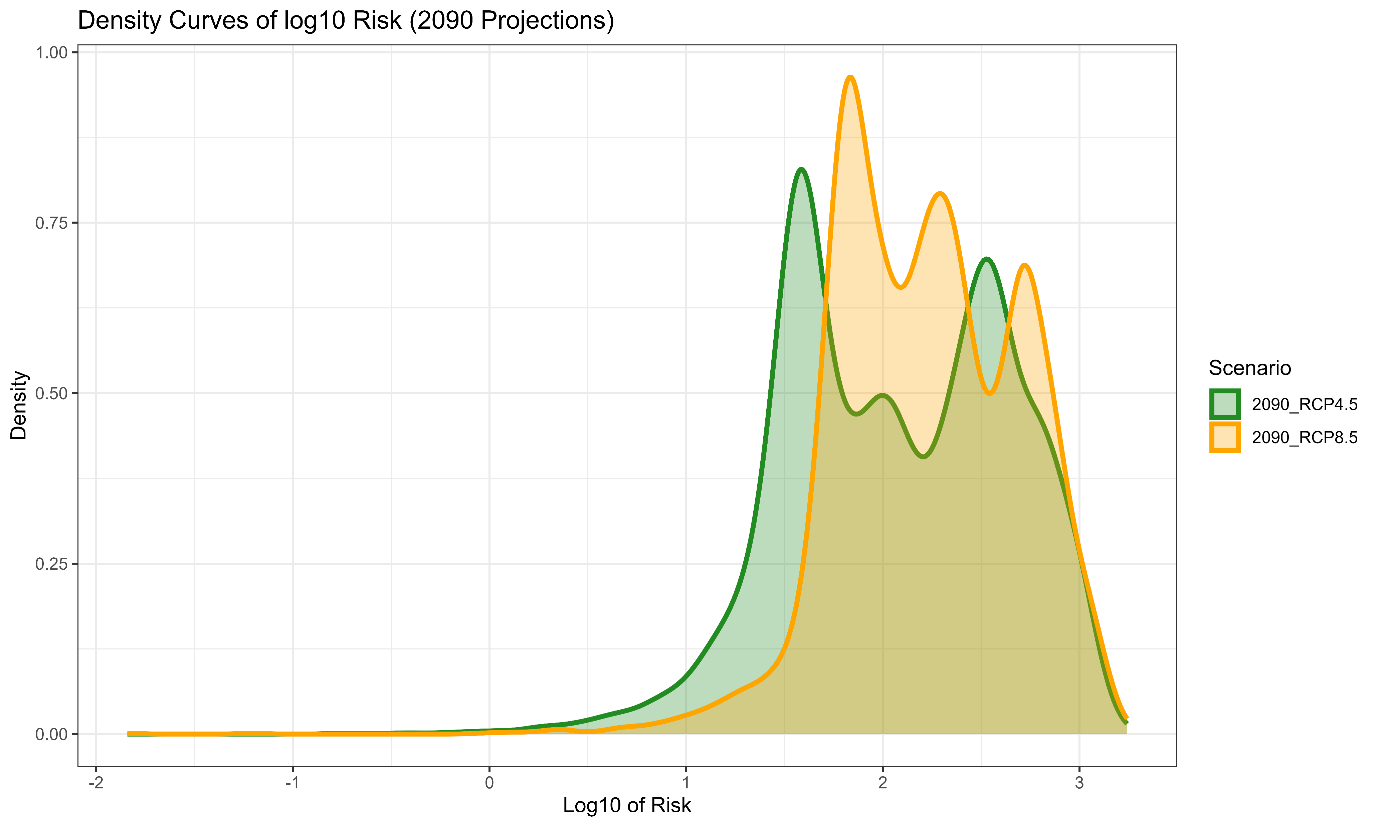


**Fig. S3.** Density plot showing the distribution of log₁₀-transformed fasciolosis risk values across Victoria under RCP 4.5 and RCP 8.5 emission scenarios for the year 2090. The x-axis represents transformed risk values while the y-axis represents the density of grid points.


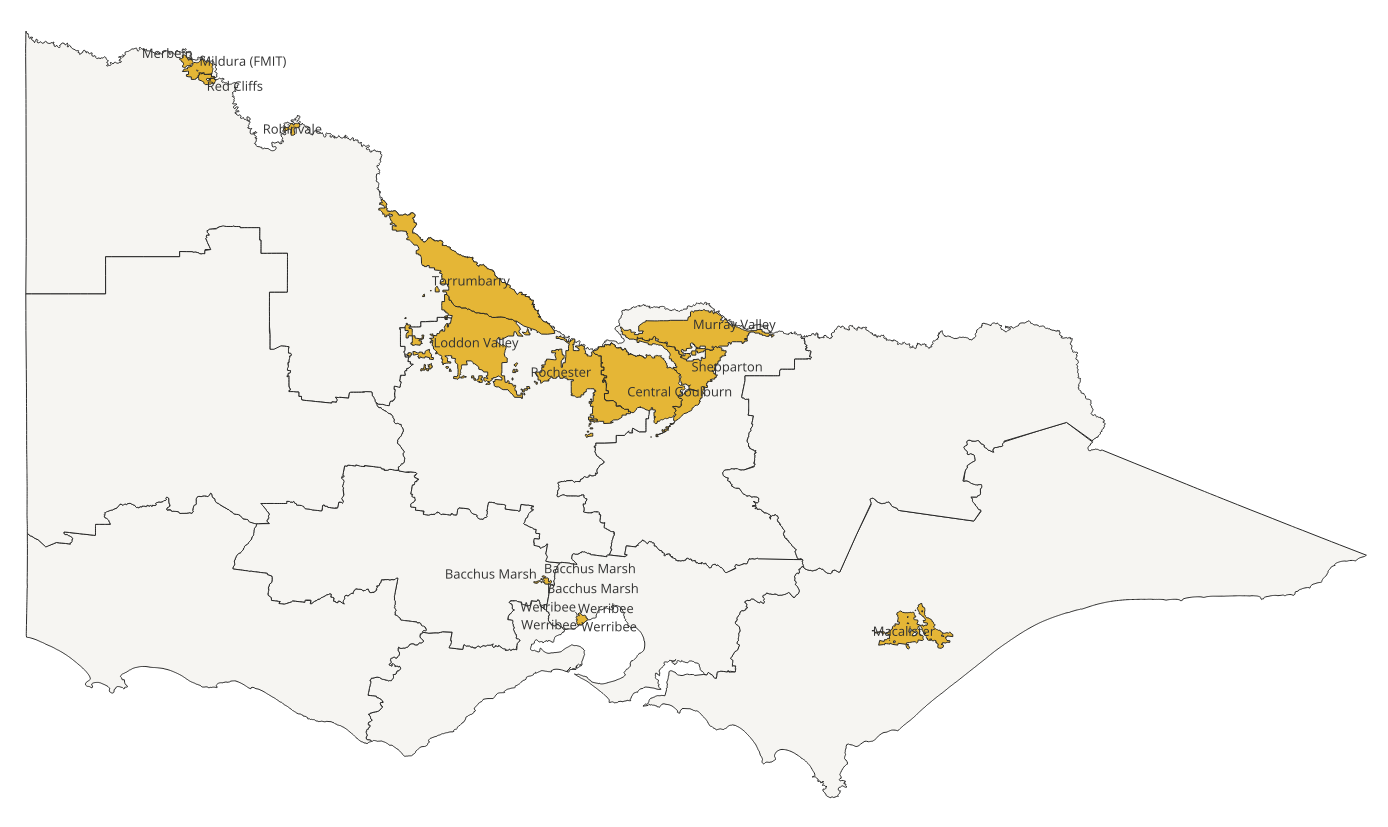


**Fig. S4.** The map illustrates the spatial distribution of officially declared irrigated regions across the state of Victoria, highlighting areas where irrigation activities are formally designated and regulated.
