# Supplementary material for: High-resolution climate modelling of fasciolosis risk in Australia: A One Health early-warning framework
Source: One Health. 2026 May 26;22:101451. doi: 10.1016/j.onehlt.2026.101451 (PMC13227242; doi:10.1016/j.onehlt.2026.101451)
Supplement: Supplementary file 3 — Supplementary material 3 [file mmc3.docx]

**Supplementary Table 2.** Past trends (1975-2024) and projected future changes of fasciolosis risk across local government areas (LGAs) of Victoria under RCP 4.5 and RCP 8.5 emission scenarios.

| **Region** | **LGA regions** | **1975-2024*** | **2050*** | | **2090*** | |
| --- | --- | --- | --- | --- | --- | --- |
|  |  |  | RCP 4.5 | RCP 8.5 | RCP 4.5 | RCP 8.5 |
| Gippsland | Bass Coast | 650 (442, 812) | 765 (660, 802) | 814 (687, 851) | 639 (603, 814) | 817 (704, 850) |
|  | Baw Baw | 142 (0, 340) | 202 (0, 358) | 250 (0, 429) | 235 (0, 348) | 485 (389, 649) |
|  | East Gippsland | 267 (43, 515) | 0 (0, 415) | 87 (0, 460) | 0 (0, 398) | 172 (0, 407) |
|  | Latrobe City | 280 (84, 429) | 136 (54, 293) | 165 (112, 259) | 171 (88, 213) | 386 (288, 480) |
|  | South Gippsland | 593 (430, 839) | 578 (430, 743) | 644 (469, 791) | 581 (417, 700) | 802 (675, 961) |
|  | Wellington | 232 (32, 394) | 23 (0, 210) | 78 (0, 284) | 54 (0, 155) | 152 (78, 222) |
| Greater Melbourne | Bayside City | 437 (250, 569) | 322 (322, 323) | 358 (358, 358) | 354 (354, 354) | 237 (237, 237) |
|  | Brimbank City | 225 (98, 333) | 52 (48, 56) | 71 (67, 74) | 51 (49, 52) | 0 (0, 0) |
|  | Cardinia | 639 (429, 782) | 464 (418, 532) | 521 (454, 588) | 447 (389, 544) | 543 (521, 747) |
|  | Casey City | 603 (414, 776) | 487 (471, 537) | 331 (313, 464) | 402 (381, 430) | 504 (348, 529) |
|  | Darebin City | 371 (181, 539) | 222 (214, 242) | 257 (253, 277) | 109 (109, 120) | 167 (166, 170) |
|  | Frankston City | 487 (284, 663) | 511 (461, 529) | 361 (351, 373) | 378 (288, 455) | 476 (386, 566) |
|  | Glen Eira City | 514 (278, 621) | 310 (310, 310) | 349 (349, 349) | 203 (203, 203) | 216 (216, 216) |
|  | Greater Dandenong City | 531 (348, 720) | 487 (363, 500) | 343 (339, 349) | 218 (210, 308) | 241 (240, 261) |
|  | Hobsons Bay City | 281 (127, 384) | 76 (72, 81) | 89 (85, 92) | 61 (59, 64) | 53 (27, 81) |
|  | Hume City | 131 (0, 263) | 0 (0, 20) | 20 (5, 43) | 27 (14, 38) | 47 (0, 93) |
|  | Kingston City | 423 (257, 635) | 319 (318, 411) | 353 (349, 353) | 206 (205, 214) | 227 (226, 240) |
|  | Knox City | 569 (377, 363) | 539 (502, 575) | 601 (515, 636) | 444 (430, 478) | 549 (539, 552) |
|  | Maroondah City | 507 (322, 622) | 542 (537, 547) | 609 (606, 613) | 495 (454, 536) | 538 (536, 539) |
|  | Melton City | 107 (19, 251) | 12 (0, 17) | 1 (0, 8) | 32 (25, 38) | 0 (0, 0) |
|  | Melbourne City | 379 (160, 525) | 110 (110, 110) | 117 (117, 117) | 76 (76, 76) | 116 (116, 116) |
|  | Mornington Peninsula | 566 (416, 724) | 759 (621, 845) | 639 (504, 701) | 587 (553, 679) | 736 (671, 813) |
|  | Nillumbik | 314 (108, 495) | 306 (246, 340) | 240 (192, 286) | 256 (203, 326) | 298 (192, 456) |
|  | Stonnington City | 445 (244, 645) | 317 (317, 317) | 134 (134, 134) | 141 (141, 141) | 208 (208, 208) |
|  | Whitehorse City | 552 (310, 692) | 468 (467, 474) | 325 (321, 332) | 317 (314, 348) | 328 (232, 453) |
|  | Whittlesea City | 126 (1, 347) | 41 (0, 84) | 76 (8, 136) | 48 (0, 90) | 149 (128, 196) |
|  | Wyndham City | 167 (54, 284) | 30 (22, 46) | 41 (2, 68) | 46 (41, 54) | 0 (0, 0) |
|  | Yarra Ranges | 0 (0, 0) | 8 (0, 359) | 140 (0, 417) | 65 (0, 428) | 514 (151, 696) |
| Ovens Murray | Alpine | 0 (0, 0) | 0 (0,0) | 0 (0,0) | 0 (0,0) | 0 (0, 132) |
|  | Benalla Rural City | 0 (0, 110) | 0 (0, 20) | 0 (0,0) | 0 (0, 3) | 98 (66, 118) |
|  | Indigo | 0 (0, 117) | 0 (0,0) | 0 (0,0) | 0 (0,0) | 80 (67, 154) |
|  | Mansfield | 0 (0, 0) | 0 (0,0) | 0 (0,0) | 0 (0,0) | 0 (0, 135) |
|  | Towong | 0 (0, 0) | 0 (0,0) | 0 (0,0) | 0 (0,0) | 0 (0, 120) |
|  | Wangaratta Rural City | 0 (0, 90) | 0 (0,0) | 0 (0,0) | 0 (0,0) | 82 (25, 139) |
|  | Wodonga City | 0 (0, 268) | 0 (0, 45) | 0 (0, 92) | 0 (0, 31) | 114 (71, 146) |
| Goulburn | Greater Shepparton City | 284 (35, 577) | 103 (71, 143) | 125 (0, 157) | 181 (27, 228) | 454 (96, 488) |
|  | Mitchell | 0 (0, 21) | 0 (0,0) | 0 (0,0) | 0 (0,0) | 60 (36, 72) |
|  | Moira | 53 (0, 139) | 8 (0, 144) | 13 (0, 145) | 16 (8, 195) | 67 (0, 184) |
|  | Murrindindi | 0 (0, 0) | 0 (0, 0) | 0 (0, 0) | 0 (0,0) | 138 (57, 231) |
|  | Strathbogie | 0 (0, 94) | 1 (0, 39) | 0 (0, 0) | 0 (0, 9) | 77 (54, 95) |
| Loddon Campaspe | Campaspe | 193 (7, 471) | 77 (0, 119) | 8 (0, 163) | 137 (18, 170) | 187 (0, 213) |
|  | Central Goldfields | 0 (0, 40) | 0 (0, 25) | 0 (0, 0) | 0 (0,0) | 53 (49, 56) |
|  | Greater Bendigo City | 0 (0, 53) | 0 (0, 0) | 0 (0, 0) | 0 (0,0) | 54 (44, 60) |
|  | Macedon Ranges | 0 (0, 0) | 0 (0, 0) | 0 (0, 0) | 0 (0,0) | 11 (0, 44) |
|  | Mount Alexander | 0 (0, 0) | 0 (0, 0) | 0 (0, 0) | 0 (0,0) | 42 (19, 48) |
|  | Loddon | 25 (0, 131) | 6 (0, 190) | 8 (0, 63) | 19 (0, 205) | 0 (0, 72) |
| Barwon | Colac Otway | 275 (106, 450) | 207 (110, 456) | 250 (145, 450) | 251 (73, 450) | 517 (285, 914) |
|  | Surf Coast | 286 (120, 388) | 213 (169, 314) | 237 (199, 331) | 169 (97, 222) | 234 (172, 461) |
| Central Highlands | Ararat Rural City | 0 (0, 0) | 0 (0, 0) | 0 (0, 0) | 0 (0,0) | 76 (62, 86) |
|  | Ballarat City | 0 (0, 0) | 0 (0, 0) | 0 (0, 0) | 0 (0,0) | 15 (7, 20) |
|  | Golden Plains | 32 (0, 144) | 1 (0, 36) | 19 (0, 38) | 29 (0, 46) | 74 (60, 89) |
|  | Hepburn | 0 (0, 0) | 0 (0, 0) | 0 (0, 0) | 0 (0,0) | 6 (0, 24) |
|  | Moorabool | 0 (0, 0) | 0 (0,0) | 0 (0, 0) | 0 (0,0) | 39 (0, 59) |
|  | Pyrenees | 0 (0, 0) | 0 (0,0) | 0 (0, 0) | 0 (0,0) | 46 (31, 57) |
| Mallee | Buloke | 14 (0, 101) | 19 (11, 28) | 0 (0, 0) | 0 (0,0) | 0 (0, 0) |
|  | Gannawarra | 256 (39, 412) | 166 (25, 267) | 180 (0, 218) | 226 (0, 265) | 182 (0, 193) |
|  | Mildura Rural City | 23 (0, 69) | 24 (0, 42) | 0 (0, 0) | 0 (0,0) | 3 (0, 0) |
|  | Swan Hill City | 28 (0, 84) | 29 (0, 37) | 0 (0, 0) | 0 (0,0) | 0 (0, 0) |
| Wimmera Southern Mallee | Hindmarsh | 13 (0, 74) | 20 (5, 24) | 0 (0, 24) | 0 (0,0) | 0 (0,0) |
|  | Horsham Rural City | 0 (0, 56) | 0 (0, 9) | 9 (0, 15) | 0 (0, 5) | 63 (0, 83) |
|  | Northern Grampians | 0 (0, 22) | 0 (0, 32) | 0 (0, 13) | 0 (0, 12) | 51 (31, 70) |
|  | West Wimmera | 34 (0, 96) | 37 (16, 76) | 36 (19, 72) | 13 (0, 46) | 83 (60, 137) |
|  | Yarriambiack | 18(0, 95) | 27 (20, 32) | 0 (0, 19) | 0 (0,0) | 0 (0,0) |
| Great South Coast | Corangamite | 170 (38, 348) | 140 (80, 442) | 170 (54, 363) | 142 (52, 333) | 387 (134, 556) |
|  | Glenelg | 248 (128, 428) | 354 (226, 469) | 394 (267, 503) | 297 (190, 391 | 539 (329, 646) |
|  | Moyne | 236 (71, 379) | 236 (94, 388) | 232 (137, 425) | 190 (115, 319) | 416 (225, 565) |
|  | Southern Grampians | 0 (0, 66) | 0 (0, 63) | 46 (0, 96) | 0 (0, 64) | 154 (103, 201) |
|  | Warrnambool City | 365 (273, 647) | 574 (557, 594) | 612 (593, 636) | 504 (419, 596) | 748 (714, 790) |

* Risk values are summarised as median with interquartile range (25^th^ and 75^th^ percentile) calculated over the baseline period (1975-2024) and in 2050 and 2090.

**Supplementary Table 3.** Past trends (1975-2024) and projected future changes in fasciolosis risk across declared irrigated districts of Victoria under RCP 4.5 and RCP 8.5 emission scenarios.

| District | 1975-2024* | 2050* | | 2090* | |
| --- | --- | --- | --- | --- | --- |
|  |  | RCP 4.5 | RCP 8.5 | RCP 4.5 | RCP 8.5 |
| Bacchus Marsh | 663 (301, 1065) | 474 (474, 474) | 533 (533, 533) | 543 (543, 543) | 221 (221, 221) |
| Central Goulburn | 390 (101, 638) | 102 (91, 111) | 125 (114, 135) | 174 (152, 192) | 456 (202, 467) |
| Loddon Valley | 285 (112, 540) | 246 (215, 273) | 223 (197, 256) | 246 (227, 260) | 363 (353, 369) |
| Macalister | 1209 (734, 1630) | 1125 (1107, 1167) | 870 (857, 900) | 906 (856, 1282) | 1021 (956, 1102) |
| Merbein | 286 (83, 475) | 130 (130, 130) | 130 (130, 130) | 117 (117, 117) | 221 (221, 221) |
| Mildura (FMIT) | 257 (104, 489) | 137 (127, 148) | 131 (127, 140) | 118 (114, 122) | 218 (197, 224) |
| Murray Valley | 442 (130, 756) | 191 (129, 241) | 177 (137, 232) | 231 (185, 260) | 464 (180, 491) |
| Red Cliffs | 201 (93, 486) | 292 (290, 295) | 137 (136, 139) | 117 (116, 119) | 218 (217, 219) |
| Robinvale | 228 (131, 536) | 232 (231, 234) | 94 (94, 95) | 29 (29, 29) | 185 (185, 185) |
| Rochester | 424 (135, 593) | 128 (116, 141) | 142 (132, 154) | 170 (156, 184) | 352 (201, 384) |
| Shepparton | 405 (116, 776) | 182 (143, 211) | 185 (138, 202) | 247 (200, 260) | 497 (291, 512) |
| Torrumbarry | 348 (116, 535) | 267 (197, 302) | 222 (196, 253) | 261 (234, 284) | 195 (190, 213) |
| Werribee | 890 (625, 1251) | 823 (823, 823) | 791 (791, 791) | 790 (790, 790) | 922 (922, 922) |

* Risk values are summarised as median with interquartile range (25^th^ and 75^th^ percentile) calculated over the baseline period (1975-2024) and in 2050 and 2090.
